# Comparative Analysis of Deep Mutational Scanning Datasets in Enteroviruses A and B Identifies Functional Divergence and Therapeutic Targets

**DOI:** 10.21203/rs.3.rs-7483105/v1

**Published:** 2025-09-08

**Authors:** Beatriz Álvarez-Rodríguez, William Bakhache, Lauren McCormick, Ron Geller, Patrick T. Dolan

**Affiliations:** 1 Institute for Integrative Systems Biology (I2SysBio), Universitat de Valencia-CSIC, Valencia, Spain; 2 Quantitative Virology and Evolution Unit, Laboratory of Viral Diseases, NIH-NIAID Division of Intramural Research, Bethesda, MD, USA; 3 Department of Biology, University of Oxford, Oxford, UK

**Keywords:** enteroviruses, enterovirus A71, coxsackievirus B3, deep mutational scanning, drug discovery

## Abstract

Deep mutational scanning (DMS) can define functional constraints acting on viral proteomes by quantifying the effects of mutations on viral fitness. However, DMS analyses do not discern type-specific from species-level constraints, limiting their utility in understanding how selective pressures change as viral families diversify. Here, we show that comparison of DMS datasets from related viruses can overcome these limitations. By contrasting two proteome-wide DMS datasets from prototypical members of the enterovirus A and B species, we identify evolutionary constraints at the species level to occur across core enzymatic machinery and capsid assembly interfaces. In contrast, type-level constraints are observed across host-interaction sites in both structural and non-structural proteins. Furthermore, we find DMS data to reflect both type- and species-level evolutionary signatures in nature yet diverge at conserved hotspots subjected to selection pressures that are lacking in vitro. Finally, we highlight the utility of comparative DMS studies for drug discovery by identifying a novel, mutationally constrained pocket in the 2C helicase that is conserved across all major human enterovirus species. Collectively, our findings provide a framework for dissecting evolutionary pressures acting at different evolutionary scales and for guiding the rational design of broad-spectrum therapeutics with high barriers to resistance.

## Introduction

Enteroviruses constitute a diverse genus of RNA viruses in the *Picornaviridae* family and include several species that pose significant and recurring public health challenges globally. Within the genus *Enterovirus* are several species that infect humans. These include the human enterovirus (HEV) A-D species, the rhinoviruses A-C, and several parechoviruses. The HEV also exhibit regional patterns of circulation, with Asian countries experiencing outbreaks of *Enterovirus A*, the USA affected by *Enterovirus D*, and Europe commonly affected by *Enterovirus B*^1^.

Initially classified by immune cross-reactivity between their capsids, and now genetically by capsid sequence, the HEV species are genetically diverse, with similarity in their polyprotein coding regions ranging from 50–60%. This diversity extends to their pathogenicity and clinical outcomes. HEV infections, which commonly affect infants, young children, and the elderly^2^, have broad tissue tropism, infecting the skin, respiratory, and gastrointestinal tissues, as well as the cardiovascular and central nervous systems (CNS). *Enterovirus A, C*, and *D* (e.g., EVA71, poliovirus, and EVD68) cause severe CNS complications such as acute flaccid myelitis and brain-stem encephalitis, which can result in paralysis and death^3^. *Enterovirus B* (e.g., coxsackievirus B3, CVB3) infection is linked with myocarditis, which can lead to cardiomyopathy and heart failure^4–6^, and pancreatic cancer development^7^.

Enteroviruses possess a single-stranded RNA genome of positive polarity of ~7.5 kb that is enclosed in a ~30 nm icosahedral particle. The viral genome is translated as a single polyprotein of ~2,150 amino acids (AA). The polyprotein is cleaved by the viral 2A protease into a structural precursor protein (P1) that encodes the capsid proteins and the nonstructural precursor (P2 and P3) that encodes the replication machinery. The P2-P3 precursor is processed further by the 3C viral protease into seven proteins (2A-2C and 3A-3D) that perform essential enzymatic and membrane remodeling activities required for viral replication^8–10^ and antagonize the innate immune system^11–13^. On the other hand, the P1 precursor is cleaved by the 3CD protease to liberate three capsid proteins, VP0, VP1, and VP3^8^. These undergo a sequential assembly to form the virus particle, starting as a heterotrimer (the “protomer”), five of which assemble to form the pentamer, and twelve pentamers form the viral capsid^14^. During maturation, VP0 undergoes an RNA-dependent autocatalytic cleavage into VP2 and VP4 to produce infectious virions. The capsid proteins of enteroviruses are highly divergent in both sequence and function, engaging with diverse host attachment and entry factors^15^. Evolutionary pressure placed on them by adaptive immunity has also driven their diversification, primarily through substitutions in surface-exposed loops^16^. Overall, mutations in structural and nonstructural proteins both contribute to the diversity of phenotypic traits observed for enteroviruses, with single non-synonymous changes having profound effects on pathogenenesis^17–20^. Thus, understanding how mutations alter viral fitness and host interaction is of key importance for understanding the biology and pathogenesis of these viruses.

With the exception of poliovirus and a regionally-approved vaccine for EVA71 in Asia, no broadly licensed vaccines exist for enteroviruses, and vaccine development is hindered by the high type diversity for each enterovirus species^21^. Similarly, while potent antivirals have been developed (e.g. Rupintrivir^22,23^), the high mutation rates^24^ of RNA viruses make antiviral resistance a major obstacle to effective therapy. The severe clinical outcomes of enterovirus infections and the lack of effective interventions underscore the urgent need for diverse therapeutic strategies. In this regard, a deeper understanding of the evolutionary mechanisms governing enterovirus fitness is critical to inform rational vaccine design and the development of antivirals with high resistance barriers.

Deep mutational scanning (DMS) is a high-throughput experimental and computational framework that enables the measurement of mutational fitness effects (MFE) for thousands of mutations in parallel. In the context of virology, DMS has been applied across different viral families to uncover functional constraints that affect viral fitness, interaction with host factors, and evasion of antibody neutralization^25–28^. As the majority of DMS studies have performed DMS on individual proteins, a direct comparison of MFEs across proteins within a viral proteome is not possible. Furthermore, the lack of DMS data for related viruses has also prevented dissecting virus-specific constraints from evolutionarily conserved pressures on a given viral family.

We recently published two full proteome DMS studies of enteroviruses EVA71 and CVB3^29,30^. Here, we leverage these datasets to develop a unified comparative framework that distinguishes between type-specific evolutionary pressures and the fundamental constraints shared by enteroviruses. Our approach identifies regions of convergent and divergent selection by directly comparing the MFE of the viral proteomes. We then integrate these MFE with structural analysis, showing that convergent constraints localize to core functional regions, including sites of capsid assembly, enzymatic activity, and membrane binding, while divergent constraints map to distinct host-factor interactions. To contextualize our findings within a real-world evolutionary framework, we compare DMS data to natural sequence variation and uncover distinct selective pressures operating in natural infections that are not captured in in vitro experimental systems. Finally, we integrate our results with computational prediction of druggable pockets to identify a novel, pan-enterovirus conserved, and mutationally constrained pocket in the viral 2C helicase, highlighting it as a high-potential target for broad-spectrum antiviral development. As more DMS studies become available, the described analysis framework can be applied to additional viral families to uncover general functional and evolutionary constraints, define sites of interaction with host proteins, and guide the rational design of novel, broad-spectrum therapeutics.

## Results

### Comparative analysis of DMS data from prototypical members of Enterovirus A and B species

**Figure 1.**
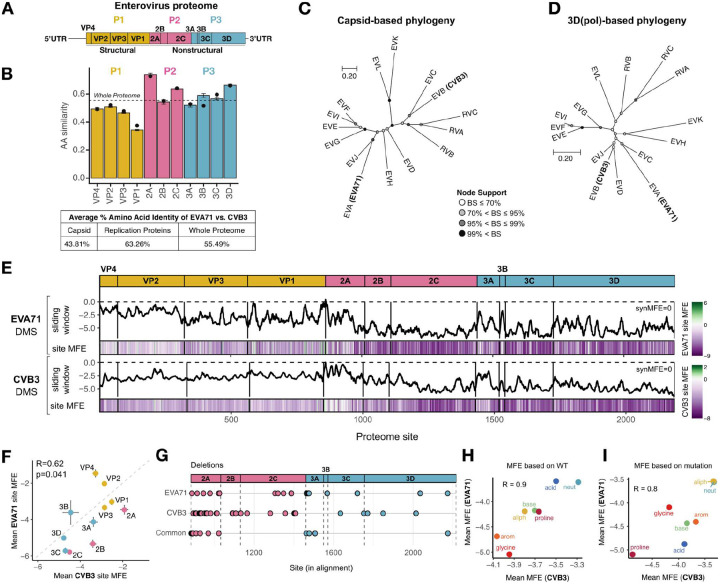
Overview of viral systems and DMS datasets. **A)** Schematic of the Enterovirus genome. **B)** Per protein similarity at the AA level between EVA71 and CVB3. **C-D)** Phylogenetic trees of all known enterovirus species, illustrating sequence divergence across structural (C) and the 3D polymerase coding regions (D). Tip labels indicate enterovirus species. **E)** Heatmap of site MFEs across EVA71 and CVB3 proteins with a 5-residue sliding window average. **F)** Correlation of mean site MFE between EVA71 and CVB3across proteins. Error bars indicate the standard error of the mean (SEM). The dashed line represents a 1:1 relationship between both axes. **G)** Genomic distribution of tolerated deletions unique for EVA71, CVB3, or shared between the two viruses (Common). **H)** Correlation of mean MFE based on the nature of the WT residue across AA physicochemical categories between the EVA71 and CVB3 DMS datasets. **I)** Correlation of mean MFE based on the nature of the mutation across AA physicochemical categories between the EVA71 and CVB3 DMS datasets.

To compare mutational tolerance across enterovirus species, we first assessed global sequence similarity and phylogenetic relationships across the proteomes of the two prototype viruses used in this study: EVA71 (Enterovirus A species) and CVB3 (Enterovirus B species) (Figure 1A). These viruses exhibit substantial divergence at the AA level, with an overall identity of ~55% across the full proteome (Figure 1B). The capsid region (P1) is the most divergent, showing only ~43% identity between EVA71 and CVB3, whereas the nonstructural region (P2–P3) shares higher similarity (~63% identity), with particularly high conservation in the 2A protease and the 3D RNA-dependent RNA polymerase.

This pattern of sequence divergence is recapitulated in phylogenetic analyses. In a tree constructed from capsid sequences (n=15), EVA71 and CVB3 form distant branches, consistent with strong antigenic and functional diversification (Figure 1C). In contrast, CVB3 and EVA71 cluster more closely in a tree based on 3D polymerase sequences (n=15), reflecting stronger evolutionary conservation of replicative functions (Figure 1D). This pattern extends beyond EVA71 and CVB3. Across different enterovirus species, capsid proteins demonstrate greater sequence divergence compared to nonstructural proteins (Supplementary Figure 2A), highlighting the distinct pressures at play on different functional regions of the proteome across various evolutionary timescales.

To dissect the shared and species-specific constraints shaping HEV evolution, we compared proteome-wide, site-specific mutational tolerance from the two published enterovirus DMS datasets, which encompass >80,000 mutations across the 11 proteins of each virus^29,30^. To enable direct comparison, we standardized both datasets using a common analysis workflow (see Methods and Supplementary Figure 1). Comparison of the standardized datasets revealed striking similarities between the two DMS datasets despite the divergence of EV A and B (Figures 1E and Supplementary figure 2A, B), as evidenced by the strong correlation of the average MFE per site (site MFEs) between the two viruses (Pearson’s R = 0.66, p < 2.2e-16, see Figure 1F for per protein MFE comparison). Specifically, capsid proteins exhibit significantly greater mutational tolerance than nonstructural proteins across both viruses. Among the nonstructural proteins, 2A and 3A are the most permissive. We also analyzed tolerance to single codon deletions, albeit only for the nonstructural region, as deletion data is unavailable for the CVB3 capsid^29^ (Supplementary figure 1). Overlapping deletion-tolerant sites between the two viruses in the 2A and 3A proteins were observed, further supporting the mutational tolerance of these two proteins (Figure 1G).

The extensive MFE datasets enabled examination of trends in structural and sequence composition that govern global mutational tolerance. Across both viruses, secondary structure context strongly influences mutational constraint, with α-helices being the most constrained structural elements, loops exhibiting the greatest mutational tolerance, and β-strands displaying intermediate tolerance levels (Supplementary figure 2D, E), highlighting the fundamental role of protein architecture in shaping evolutionary constraints across enteroviral proteomes. We also observe strong similarities in mutation tolerance based on the nature of the wild-type (WT) or mutant AA between the datasets. Specifically, averaging MFEs based on WT AA classes (e.g. aromatic, acidic, etc.), we observe consistent mutation tolerance profiles between the two datasets (Pearson’s R = 0.9, p = 0.0056; see Figure 1H). In particular, glycine and aromatic residues are among the least tolerant to substitutions, likely reflecting their unique structural roles, whereas neutral AA demonstrate the greatest mutational flexibility. Similarly, MFEs based on the nature of the mutant AA are strongly correlated between both viruses (Pearson’s R = 0.8, p = 0.034), with substitutions involving aliphatic and neutral residues being generally well tolerated, while mutations introducing proline or charged residues are predominantly deleterious across both viruses (Figure 1I). Moreover, mutations that maintain physicochemical similarity between AA are consistently the most tolerated (Supplementary Figure 2F, G). Interestingly, the impact of AA substitutions depends not only on the nature of the change but also on its directionality, reflecting fundamental constraints shaped by biochemical compatibility across proteins (Supplementary Figure 2F, G).

### A Structural Map of Evolutionary Constraints in EVA71 and CVB3

**Figure 2.**
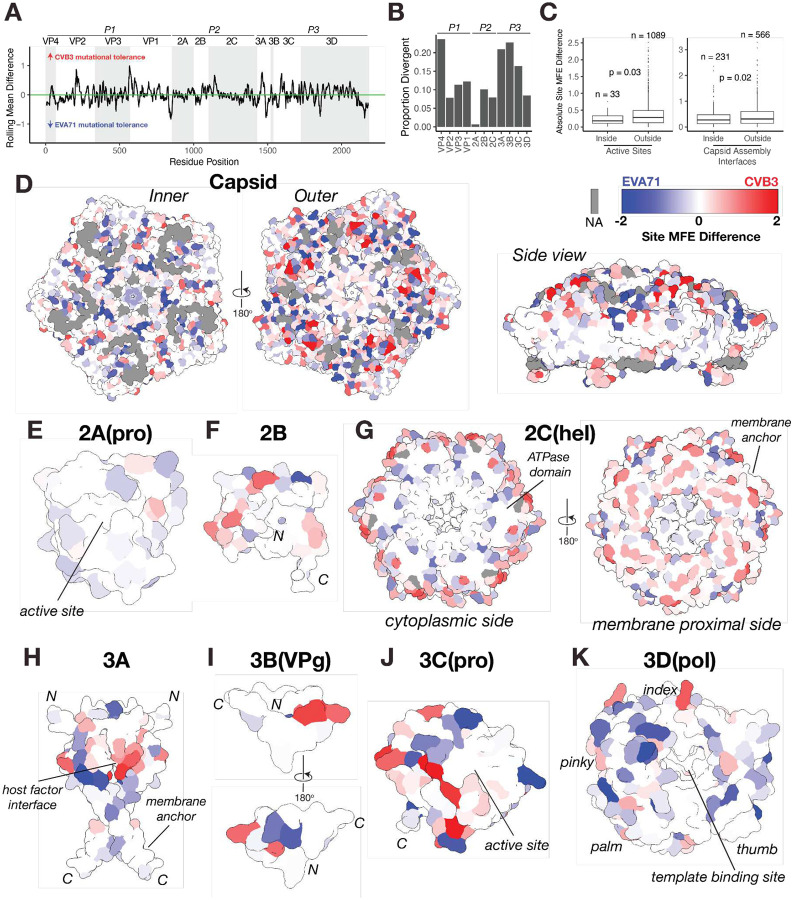
Structural mapping of Enterovirus A and B differential mutational tolerance. **A)** Line plot of the site difference in mutational fitness effect (MFE) scores (MFE in CVB3 – MFE in EVA71) across the aligned viral proteomes, averaged using a 10-residue sliding window. Positive values indicate higher mutational tolerance in CVB3, while negative values indicate higher tolerance in EVA71. A horizontal green line at y=0 denotes similar tolerance across both viruses. **B)** Bar plot quantifying the proportion of divergent sites for each viral protein. Divergent sites are defined as those with an absolute site MFE difference greater than the median plus two median absolute deviations (threshold = 0.86). Proportions are calculated relative to the total number of aligned residues per protein. **C)** Box plot showing the distribution of absolute site MFE difference for sites located inside versus outside active sites and capsid assembly interfaces. Statistical significance was calculated using a Wilcoxon test. **D-K)** Structural mapping of site MFE difference on the structure of the (D) capsid pentamer (PDB:8E2X), (E) 2A protease, (F) 2B protein, (G) 2C helicase (hexameric form), (H) 3A protein (dimeric form), (I) 3B protein (VPg), (J) 3C protease, and (K) 3D polymerase. All models are based on the EVA71 proteins (from experimental structures or AlphaFold3 predictions), which serve as a representative scaffold. Surfaces are colored by the site MFE difference according to the key: red indicates higher tolerance in CVB3, blue indicates higher tolerance in EVA71, and white represents sites under convergent constraint (similar MFE scores). Key functional regions are labeled, and gray denotes positions with missing data due to structural alignment gaps.

Next, to place the evolutionary constraints between EVA71 and CVB3 in a functional context, we calculated the site-wise differences in mean MFE and defined divergent sites as those exceeding the median plus two median absolute deviation (see Methods; Figure 2A). This analysis reveals that VP4, 3A, and 3B have the greatest proportion of sites with divergent fitness effects between the two viruses. In contrast, the 2A protease shows the lowest proportion of divergent sites, indicating similar mutational flexibility in both viruses (Figure 2B).

To understand the structural basis for these differences, we mapped site-wise divergence values onto the viral protein structures. Generally, sites in enzymatic active sites or those participating in capsid assembly are significantly more conserved than sites outside of these regions (Figure 2C). This analysis further highlights regions of strong convergent constraint corresponding to common essential functions, including the interfaces between capsid pentamers that are important for virion assembly (Figure 2D, side view), the catalytic sites of the 2A and 3C proteases (Figure 2E and J), the ATPase domain of the 2C helicase (Figure 2G, cytoplasmic view), and the template-binding channel of the 3D polymerase (Figure 2K). In contrast, regions known to mediate host-factor interactions are dominated by divergent mutational tolerance. This is most evident on the exterior surface of the viral capsid, where large patches corresponding to surface-exposed loops were substantially more tolerant to mutation in one virus than the other (Figure 2D, outer view). These loops are primary targets for the host adaptive immune system^16,31^ and contain determinants for virus-specific receptor binding^20,32^, explaining the strong type-specific selective pressures. A similar pattern of divergence occurs in the N-terminal region of the 3A protein, which harbors a known host-factor binding interface^33,34^ (Figure 2H).

### Divergent Mutational Tolerance at the Receptor-Binding Footprints of Enterovirus Capsids

**Figure 3.**
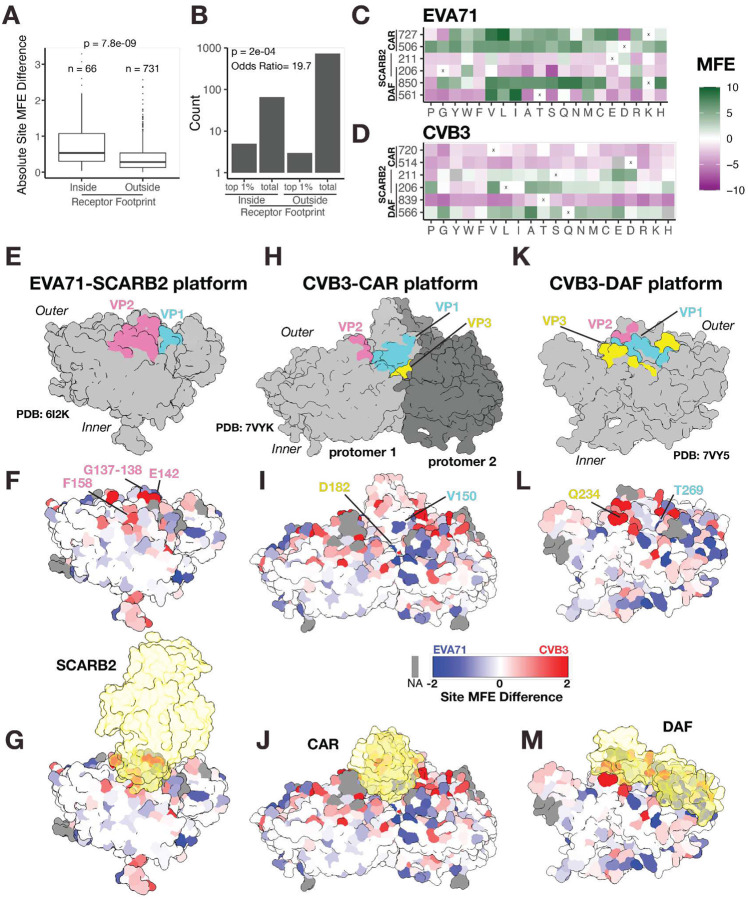
Divergence in mutational tolerance at the EVA71 and CVB3 receptor-binding footprints. **A)** Box plot showing the distribution of absolute site MFE difference for sites located inside versus outside receptor footprints. Statistical significance was calculated using a Wilcoxon test. **B)** Bar plot showing the total number of analyzed sites alongside the count of the most divergent sites (top 1% by MFE difference), categorized by their location inside or outside the receptor binding interface. Statistical significance was calculated using a Fisher’s exact test. **C-D)** Heatmap displaying the EVA71 (C) and CVB3 (D) MFE scores for divergent residues in receptor contact sites. The “X” symbol indicates WT AA position, and gray squares represent missing data. **E-M)** Surface representations of the EVA71 protomer in complex with its receptors, SCARB2 (PDB: 6I2K) (E-G), CAR (PDB: 7VYK) (H-J), or DAF (PDB: 7VY5) (K-M). In (E), (H) and (K), contact loops on the protomer are highlighted: VP1 (cyan) and VP2 (pink) and VP3 (yellow). In (F,G, I, J, L, and M), the protomer surface is colored by the site MFE difference between EVA71 and CVB3. In (G, J, and M), the receptor molecules are shown as a transparent yellow surface representation. For structural models (F, G, I, J, L, M), surfaces are colored according to the key: red indicates higher mutational tolerance in CVB3, while blue indicates higher tolerance in EVA71. White denotes sites of convergent constraint. Gray denotes positions with missing data due to structural alignment gaps.

Given that EVA71 and CVB3 use different host entry factors, Scavenger receptor class B member 2 (SCARB2)^32,35^ for EVA71 and Coxsackie- and Adenovirus Receptor (CAR) and Decay-Accelerating Factor (DAF; CD55) for CVB3^20^, we hypothesize that structural divergence on the capsid surface would map to these receptor-binding footprints. Indeed, our analysis shows that site-wise MFE divergence is significantly enriched within these footprints compared to other capsid regions (Figure 3A). Notably, ~60% of the most divergent sites (top 1%) are located within a receptor-binding region, making a highly divergent site nearly 20 times more likely to be found in this region (Figure 3B). This pattern reflects a functional constraint in which interaction interfaces with a cognate receptor limit mutation tolerance of an otherwise variable region in viruses employing other entry factors (Figure 3C, D).

To visualize these results, we map the site MFE differences in the context of the known structure of the EVA71 capsid in complex with its receptor, SCARB2 (PDB: 6I2K). The structure shows SCARB2 binding to the southern rim of the viral canyon through contacts in the VP1 GH and VP2 EF loops (Figure 3E). These specific receptor-binding sites are under strong mutational constraint in EVA71 but tolerant to mutation in CVB3 (Figure 3C–D, 3F–G). This divergence is driven by the residues G137-G138, E142, and F158 in the VP2 EF loop. The same structural view also shows that capsid assembly interfaces are under strong convergent constraint (white regions), supporting the specificity of the divergence at the receptor footprint (Figure 3F, G).

In the reciprocal analysis, we map the site MFE differences onto the structure of CVB3 in complex with its principal entry receptor, CAR (PDB: 7VYK). CAR binds within the CVB3 canyon at an interface involving the VP1, VP2, and VP3 capsid proteins (Figure 3H). The VP2 CAR-binding region involves an inserted sequence, resulting in a local structural rearrangement in CVB3, relative to EVA71, that accommodates CAR binding. Consequently, comparing constraints at all sites in the footprint is not possible. Nonetheless, the canyon region generally exhibits strong mutational constraint, and we observe clear divergence at the CAR contact residues V150 in VP1 and D182 in VP3, which are under stronger mutational constraint in CVB3 than in EVA71 (Figure 3D, 3H–I).

Counter-intuitively, a more complex pattern emerges when we analyze the interaction sites for the CVB3 attachment factor, DAF (PDB: 7VY5). Some residues interacting with DAF show higher mutational tolerance in CVB3 than in EVA71, the opposite of the expected trend for a functional binding site (Figure 3C, D, K–M). This unexpected tolerance is particularly notable at residue Q234 in VP3. Interestingly, this residue has been identified as a key “molecular switch” that modulates the ability of the virus to shift from DAF-dependent to DAF-independent entry pathways^20,29^, providing a potential explanation for this biological phenomenon. Alternatively, the high expression of CAR on HeLa-H1 cells^16^ may reduce dependency on DAF entry, alleviating structural constraints pertaining to the use of this receptor.

### Membrane and Host-Factor Interactions Drive Enterovirus 3A Mutational Divergence

**Figure 4.**
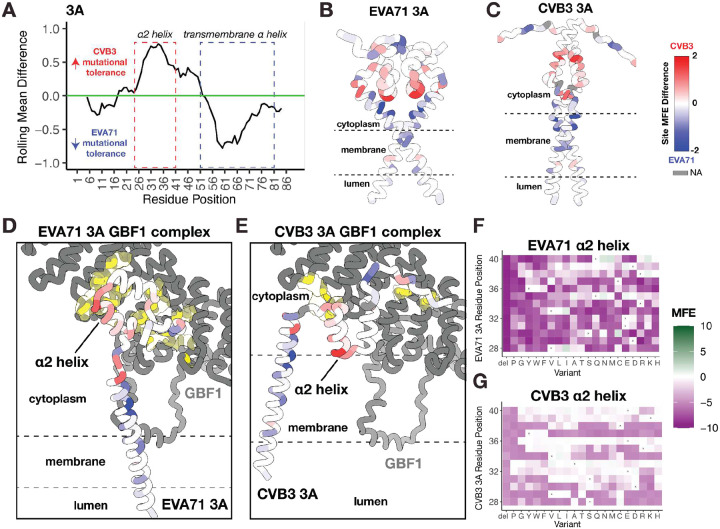
Mapping of the divergent structural and functional regions in Enterovirus 3A. **A)** Line plot of the site difference in mutational fitness effect (MFE) scores (MFE in CVB3 - MFE in EVA71) across the aligned 3A proteins, averaged using a 10-residue sliding window. **B-C)** AlphaFold3 predicted structures of the EVA71 (B) and CVB3 (C) 3A homodimers. Residues are colored by site MFE difference according to the color key. **D-E)** Predicted structural models of the EVA71 (D) and CVB3 (E) 3A dimers in complex with the host factor GBF1, as determined by AlphaFold3. GBF1 is shown as a gray surface, with its 3A contact residues highlighted in yellow. **F-G)** Heatmaps displaying MFE scores for the specific set of residues that are both mutationally divergent and that constitute the EVA71-GBF1 binding interface. Data are shown for EVA71 (F) and CVB3 (G). X indicates WT AA position. For all relevant panels, predicted protein topology (cytoplasm, membrane, lumen), as determined by DeepTMHMM, is indicated. For all structural views, a common color key is used for the MFE difference: red indicates higher tolerance in CVB3, blue indicates higher tolerance in EVA71, and white denotes sites of convergent constraint. Gray denotes positions with missing data due to structural alignment gaps.

The divergent mutational tolerance we observe between the EVA71 and CVB3 3A proteins suggests that they engage host factors through distinct interfaces (Figure 4A). The enterovirus 3A proteins of both viruses bind host membranes to facilitate the formation of viral replication organelles^10^. Additionally, CVB3 3A blocks protein trafficking in the cell via interaction with GBF1^36^, while EVA71 does not, despite also interacting with this host factor^37^. Hence, we hypothesize that these differences could be explained by distinct interactions with both the lipid membrane and the host protein GBF1.

We first used DeepTMHMM^38^ to predict the topology of the 3A proteins in order to investigate the 3A membrane-spanning region. The software predicts a single α-helical transmembrane helix for both proteins, but with different lengths of the transmembrane region, suggesting CVB3 3A inserts deeper into the membrane than EVA71 3A. This prediction aligns with our DMS data, which show that the membrane-embedded region in CVB3 is mutationally constrained, whereas the corresponding region in EVA71 3A is predicted to lie outside the membrane and is tolerant to substitutions (Figure 4B–C, Supplementary Figure 3). While this suggests that distinct protein-lipid interactions impose unique selective pressures on each virus, further biochemical work is needed to fully characterize the differences in mutational flexibility we observe in the transmembrane domain of enterovirus 3A.

Second, to examine potential differences in the interaction with GBF1, we use AlphaFold3 to model the 3A dimer from both viruses in complex with the complete form of this host factor (Figure 4D, E, Supplementary Figure 4). Again, our models predict a difference in this virus-host interaction interface. In EVA71, GBF1 makes contact with both the α1 and α2 helices of the 3A dimer. In contrast, the corresponding residues on the CVB3 3A dimer are oriented away from GBF1 and not involved in the binding interface. This region also exhibits greater mutational tolerance, consistent with the distinct predicted binding modes in CVB3 and EVA71 3A (Figure 4 F–G). Moreover, these findings are also congruent with published biochemical data and computational work suggesting the importance of the N-terminal region in mediating interaction with GBF1 interaction and COP-I transport^33,34^. While our predictions explain differences in MFEs between EVA71 and CVB3 3A, more structural studies are needed to map the distinct interaction surfaces between enterovirus 3A proteins and GBF1.

### Comparison of laboratory and natural selection pressures

Having established conserved and virus-specific patterns of mutational tolerance across the two experimental DMS datasets, we next assess how these trends relate to natural sequence variation. We use the phydms software^39^ to incorporate experimentally measured MFEs into phylogenetic models derived from natural sequence alignments across two evolutionary scales - type (EVA71, CVB3) and species (Enterovirus A, Enterovirus B). In all cases, incorporation of MFE information into phylogenetic models outperforms standard models (YNGKP/Goldman-Yang models) (Supplementary Table 1). These observations suggest that DMS-derived MFEs reflect selection processes operating in nature across both evolutionary timescales.

**Figure 5.**
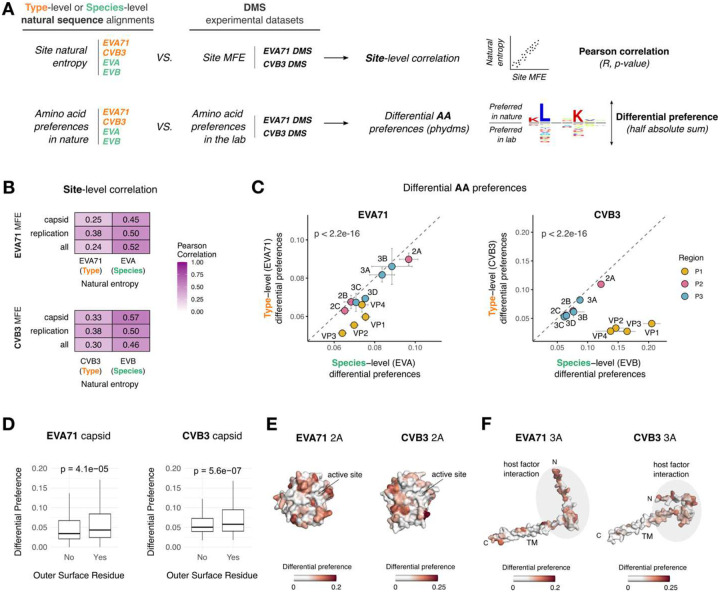
Comparison of laboratory and natural selection pressures. **A)** Workflow used for the comparison of natural diversity and DMS-derived MFEs. Sequence alignments were used to obtain a site-level diversity measure (Shannon entropy) and AA preferences. These metrics were compared with site MFEs and AA preferences derived from the DMS datasets. Site-level analyses were performed using Pearson correlation tests, and differential AA preferences between nature and laboratory were obtained using the phydms software. **B)** Correlation between experimental DMS datasets and natural sequence diversity at the species and type levels, calculated separately for different functional regions (capsid, replication, or whole-proteome). All Pearson correlations shown are statistically significant (p-value<0.05). **C)** Differential AA preferences per protein between experimental DMS datasets and natural sequence diversity for CVB3 and EVA71 at the species and type levels. Statistical significance was calculated using a Wilcoxon test (species vs type). The dashed line represents a 1:1 relationship between both axes. **D)** Differential AA preferences between DMS datasets and natural sequences at the type level in surface-exposed versus buried capsid residues for EVA71 and CVB3. **E-F)** Structural mapping of differential preferences between DMS datasets and natural sequences at the type level onto the 2A (E) and 3A (F) proteins for EVA71 and CVB3. TM, trans-membrane. Host factor interaction regions in 3A include residues involved in ACBD3 binding (EVA71 3A), GBF1 binding, membrane trafficking disruption, and recruitment of PI4KIIIβ (CVB3 3A).

To further explore the evolutionary signatures captured by our datasets, we analyze the correlation between MFEs and a measure of variation in natural sequence alignments across both evolutionary scales, Shannon entropy (H; Figure 5A). This revealed that DMS-derived mutational tolerance correlates more strongly with site entropy at the species level (Pearson’s R = 0.52, p = 6.84e-150; R = 0.46 p = 3.99e-114; for EV-A and EV-B, respectively) than at the type level (Pearson’s R = 0.24, p = 7.49e-31; R = 0.3, p = 2.34e-46; for EVA71 and CVB3, respectively; Figure 5B). This effect is primarily driven by differences in the variability of the structural proteins, which show strong conservation at the type level but high variability at the species level, in agreement with MFE data (Supplementary figure 5A, B). Moreover, DMS-derived MFEs correlate more strongly with site entropy from other enterovirus species than with variation within their own types (Supplementary figure 5C, D). Together, these findings reveal DMS to capture broader patterns of capsid constraints that operate at longer evolutionary timescales and are conserved across different enterovirus species.

To further identify differences between selection pressures in the lab and nature, we examined whether the same AA were preferred across each site between lab-derived MFE and natural sequences at both the type and species level. For this, we used the phydms software to calculate differential preference scores for each site, with larger values indicating greater discordance between AA preferred in DMS and nature (Figure 5A). In contrast to the observation that site-wise mutational tolerance aligns more closely with natural diversity at the species level (Figure 5B), greater concordance with natural variation at the type level was observed for AA preferences for both viruses (p < 2.2e-16 for type vs. species by Wilcoxon test for both EVA71 and CVB3; Figure 5C). This pattern is again primarily driven by the capsid proteins (Supplementary figure 5E). Notably, although overall similar AA were preferred across the capsid in both the DMS results and sequence alignments at the type level, sites showing larger differences in preferred AA were specifically enriched in surface-exposed residues in both viruses (p = 4.1e-05 and 5.6e-07 for EVA71 and CVB3, respectively, by Wilcoxon test; Figure 5D). This likely reflects host interactions not captured in our *in vitro* DMS experiments. Indeed, for CVB3, where we have previously comprehensively defined neutralizing antibody binding sites in human sera^13^, we find differences in AA preferences to be significantly enriched in sites targeted by neutralizing antibodies compared to other surface residues (p = 4.7e-04 by Wilcoxon test; Supplementary Figure 3F).

In contrast to the structural proteins, AA preferences in the nonstructural proteins are consistent across the different evolutionary timescales, with the proteins 2A and 3A exhibiting the greatest divergence from natural AA preferences (Figures 5E, 5F). These discrepancies likely reflect natural selective pressures that are absent in our in vitro culture systems. Differences in 2A predominantly map to surface-exposed residues (p = 0.3 and p = 0.0012 for EVA71 and CVB3, respectively, by Wilcoxon test; Supplementary Figure 5), which are likely involved in interactions with host factors^35^. Although 2A is dispensable for replication in cell culture^36,37,^ it plays a key role in antagonizing the cellular type I interferon (IFN-I) antiviral response^40^. The lack of IFN-I activation in our experimental system likely explains some of the discrepancies observed between the DMS data and patterns of natural selection (Figure 5E). Similarly, for 3A, differential selection clusters in the N-terminal region for both viruses (p = 0.0098 and p = 4.4×10^−6^ for EVA71 and CVB3, respectively, by Wilcoxon test for the N-terminal domain versus membrane-binding domain; Supplementary Figure 5), which mediates interactions with host factors to promote membrane remodeling and disrupt intracellular trafficking (Figure 5F)^41^. Hence, comparison of AA preferences between DMS and sequence alignments can help uncover regions that are under selection pressures not present in vitro across both the structural and non-structural regions.

### Druggable Pocket Screen Identifies a Mutationally Constrained Target in the Enterovirus 2C helicase

**Figure 6.**
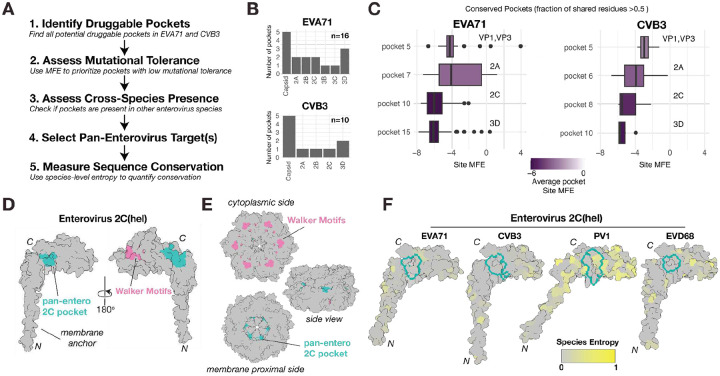
Identification of a conserved druggable pocket in the enterovirus 2C helicase. **(A)** Schematic showing the workflow for identifying and prioritizing druggable pockets, combining computational druggable pocket screening with experimental determination of mutation tolerance from DMS studies. **(B)** Bar plots showing the total number of druggable pockets detected in the EVA71 and CVB3 proteomes using the SiteMap software. **(C)** Boxplots comparing the average MFE for residues within conserved druggable pockets (fraction of shared residues > 0.5). Lower MFE scores indicate higher mutational constraint. **(D)** Surface representation of the AlphaFold3 predicted EVA71 2C helicase monomer. The identified pan-enterovirus druggable pocket (green) is shown in relation to the ATPase domain, which includes the Walker A, B, and C motifs (pink). **(E)** Surface representation of the AlphaFold3 predicted EVA71 2C helicase in its hexameric form, shown from the cytoplasmic, membrane, and side views. **(F)** Multi-species structural comparison of the 2C pocket. The surface of the AlphaFold3 predicted 2C protein from four representative enterovirus species is shown: EVA71, CVB3, PV1, and EVD68. Surfaces are colored by the natural sequence entropy at each position within their respective species, with gray indicating high conservation (low entropy) and yellow indicating high variability. The prioritized pocket region is outlined in green.

Building on our structural and functional analyses, we next explore whether our cross-species mutational tolerance data can be used to identify potential antiviral targets with high barriers to resistance (Figure 6A). Specifically, we aimed to identify druggable pockets that are intolerant to mutations such that mutations required to bypass drug binding would come at a high fitness cost for the virus and thus reduce its ability to spread in the host. For this, we first perform a computational screen for druggable pockets in the EVA71 and CVB3 proteomes using the SiteMap software^42^, which identifies and scores protein pockets based on size, geometry, and physicochemical properties that are favorable for drug binding. This screen identified 16 and 10 candidate pockets in EVA71 and CVB3, respectively (Figure 6B). Of these, four pockets were selected due to their conservation between EVA71 and CVB3 (fraction of shared residues relative to the smallest pocket >0.5; Figure 6C and supplementary figure 6A). We then apply our DMS data as a functional filter to prioritize pockets located in regions of high mutational constraint, i.e. where potential escape mutants are likely to be lethal or have significantly reduced fitness. This analysis reveals that pockets within structural proteins are generally mutationally tolerant, whereas those in nonstructural proteins are highly constrained, particularly those in the 2C and 3D proteins, making them ideal candidates for antiviral targeting (Figure 6C).

To assess the potential of these two pockets as pan-enterovirus targets, we expand our drug pocket analysis in 2C and 3D to representative species from Enterovirus C (poliovirus 1; PV1) and Enterovirus D (enterovirus D68; EVD68). To define conserved drug pockets, we calculated the fraction of shared residues in each pocket across the viruses, utilizing the smallest drug pocket as reference (Supplementary Figure 6B). The 3D pocket shows significant variation between viruses, sharing few or no residues across all four viruses (Supplementary Figure 6B). In contrast, the 2C pocket shows strong conservation across all four viruses, with at least one pocket in the 2C protein of each virus sharing >0.8 of their residues with that of CVB3 (Supplementary Figure 6B). These pockets in 2C share nine conserved residues, five of which are identical across all four viruses (Supplementary figure 6C, D). Structurally, the pocket is situated away from the well-characterized ATPase Walker motifs (Figure 6D, E) and the architecture of the pocket is preserved across all four enterovirus species, with root mean square deviation (RMSD) values of the shared residues relative to the CVB3 2C pocket ranging from 0.165 to 0.204 Å (Supplementary Figure 6F). Finally, to validate the conservation of these 9 conserved drug pocket residues in nature, we examined their variability compared to the remaining sites in the proteome across large alignments of natural sequences from each virus species (n=1081, 762, 292, and 71 for enterovirus A, B, C, and D, respectively). Overall, these residues showed lower conservation compared to the remainder of the proteome across the four enterovirus species (p < 0.05 for enterovirus B-D and p = 0.2 for enterovirus A by Wilcoxon test; Figure 6F and Supplementary Figure 6C). The high structural conservation, low natural variability, and strong fitness costs in cell culture of the residues comprising this core drug pocket support its potential as a promising target for drug development.

## Discussion

Understanding the functional consequences of mutations is a fundamental goal of evolutionary virology, holding important implications in molecular epidemiology and the design of successful therapeutics. Systematic derivation of mutational fitness effects via DMS has proven to be a powerful tool for this purpose, allowing for the comprehensive mapping of MFEs across viral proteins and the definition of sites affecting interaction with host factors, including antiviral proteins, receptors, and neutralizing antibodies^16,26,27,43^. However, the lack of DMS studies for related viruses has hindered the use of DMS to address how evolutionary constraints on viral proteomes change over longer evolutionary timescales, as viral families diversify phenotypically. Capitalizing on the recent publication of the first two proteome-wide DMS studies, we herein performed the first direct, comparative fitness mapping of two human enteroviruses belonging to two different species, EVA71 and CVB3, in order to functionally distinguish the core constraints shared between species from type-specific adaptations. Although these viruses share only ~50% sequence identity, we find that trends in mutational fitness effects are largely consistent across their proteomes (Figures 1 and 2), highlighting a core set of biophysical and functional constraints shared across species. These shared constraints map to catalytic sites (i.e. proteases, polymerase, helicase) and capsid assembly interfaces, likely representing the conserved functional core for the major human enterovirus group.

Beyond this conserved core, we identify a limited number of of sites that show divergence in their mutation tolerance in the two viruses, revealing type-specific adaptations. In the capsid proteins, distinct constraints in the two viruses are observed across sites that interact with the cognate receptor of each virus, SCARB2 for EVA71 and CAR for CVB3 (Figures 2 and 3), providing a clear type-specific virus-host interaction interface. Interestingly, we also observe divergence in constraints underlying functions of the nonstructural proteins (Figures 2 and 4). For example, divergence in mutational tolerance is observed in 3A at residues implicated in binding the host GBF1 protein (Figure 4), suggesting EVA71 and CVB3 have tuned their interaction with this critical host factor to their specific replication requirements. This phenomenon is reminiscent of the distinct membrane interactions and binding modes observed for the host factor ACBD3 by 3A proteins from distinct genera of picornaviruses: enterovirus and kobuvirus^44^. Overall, our results underscore the utility of comparative DMS analyses in uncovering type-specific host-interaction interfaces, which can help uncover the mechanisms underlying the distinct tissue tropism and pathogenic outcomes of infections with related viruses.

By integrating DMS data with natural sequence variation across viral types and species, we provide the first comprehensive analysis linking experimentally derived MFEs to evolutionary patterns across multiple evolutionary scales. Interestingly, site-wise mutational tolerance inferred from DMS correlates more strongly with broader species-level mutational flexibility than with within-type variation (Figure 5), even when comparing to different enterovirus species. Our analyses specifically identify the capsid as the main driver of the correlation with species-level diversity (Figures 5B–D). The fact that MFEs across the capsid correlate with variability at the species level and not the type level highlights the ability of DMS to capture broad structural and functional constraints that are observed in nature only when considering diverse viruses with unique receptor and immune pressures. In contrast, when examining which specific AAs are preferred at each site, DMS better reflects type-specific evolutionary constraints rather than those acting at the species level (Supplementary Figure 5E–H). This is consistent with findings in influenza hemagglutinin, where DMS profiles correspond better to natural variation within a strain than across strains^45^. Hence, our findings reveal DMS to reflect mutation tolerance at the species level but AA preference at the type level.

While DMS provides a high-resolution map of functional constraints under controlled, *in vitro* conditions, natural evolution unfolds in more complex environments that integrate diverse and dynamic host-specific pressures, including immune responses, tissue-specific replication constraints, and other selective forces largely absent from cell culture. The limited differences we observe at the type-level in the capsid proteins are enriched in surface-exposed residues involved in immune evasion. At these sites, natural sequence divergence is not fully captured by DMS, underscoring the role of host-driven immune selection in shaping these regions, consistent with prior DMS studies of viral structural proteins^46^. In contrast, nonstructural proteins exhibit strong consistency between experimental and natural constraints, and at different evolutionary scales (type and species level), reflecting their conserved functions in viral replication and intracellular processes across experimental and natural environments.

Exceptions to these trends in the nonstructural proteins are 2A and 3A, which exhibit a high mutational tolerance in the DMS datasets and large discrepancies from natural sequences in AA preferences. The elevated tolerance of 2A in vitro aligns with previous findings that this protein is dispensable for replication in cell culture, despite its essential role in antagonizing the host interferon response^36,37.^ This immune pressure is absent in the permissive in vitro environment where the DMS data were generated, likely explaining the increased mutational flexibility of 2A in this context. For 3A, differential AA preferences are concentrated in the N-terminal region of the protein across both viruses, which is known to interact with multiple host factors. Although the precise drivers of this divergence remain unclear, mutations in these regions have been shown to impair the ability of the virus to block host immune responses (antiviral RNA interference responses in EVA71^42^ and antigen presentation in CVB3^43^), which are lacking in our in vitro models. These observations support the idea that key immune-related functions of 2A and 3A are not fully captured in our experimental system, leading to discrepancies between in vitro mutational tolerance and natural evolutionary constraints. Although EVA71 and CVB3 may encounter distinct host factors and immune pressures in their respective natural contexts, the regions where in vitro and in vivo constraints most strongly diverge converge on the same proteins (the capsid, 2A, and 3A), implicating these as key hotspots of immune-driven selection across enteroviruses.

Drug resistance remains a major obstacle to efficacious antiviral therapy. Recently, DMS data has been utilized to help prioritize drug targets where mutations conferring reduced drug binding are likely to come at a high cost to viral replication, preventing the spread of resistant variants^29,47^. Herein, we integrated MFE data across the full proteome of both enteroviruses with computational druggable pocket prediction to identify such drug targets. We identify multiple shared drug pockets across the structural and non-structural regions (Fig. 6). In general, druggable pockets in structural regions showed higher mutational tolerance, in agreement with prior experimental findings from different capsid-binding antivirals (e.g. WIN compounds^48^). In the non-structural proteins, we identify a novel drug pocket in 2C that is both mutationally intolerant and structurally conserved in both viruses (Figure 6C). Utilizing natural sequence variation, we further show this drug pocket to be conserved and invariable across all major human enterovirus species (Enterovirus A-D; Figure 6F). The strong structural and sequence conservation of this drug pocket support it as a promising candidate for future development of pan-enterovirus antivirals.

Overall, these findings highlight the power of integrating DMS data from related viruses with structural and evolutionary analyses to uncover both general and specific constraints acting on viral proteomes. This analysis framework can be readily extended to additional viral families and similarly applied to discovering hotspots of type-specific host interactions, conserved druggable pockets, and both converged and divergent selection pressures.

## Methods

### Calculation of MFEs

Detailed information on the production of DMS plasmid libraries and viral populations can be found in their corresponding publications^29,30^. For libraries generated using the synthetic biology approach (P2 and P3 for CVB3 and EVA71 datasets), codon tables were filtered to keep only codons where engineered mutations were originally introduced. For the P1 libraries of CVB3, generated via PCR-based mutagenesis, all single mutations within codons were excluded from the analysis to increase the signal-to-noise ratio^49^. Mutational fitness effects (MFEs) were calculated following the general procedure described in dms_tools2 for the ratio method^50^ using custom scripts (https://github.com/QVEU/EV_DMS_Comparison). Briefly, for each site, the sum of all codons giving rise to a particular AA mutation was divided by that of the WT AA to obtain the relative enrichment of each mutation at each site. The relative enrichment for a given mutation in the viral populations was divided by its relative enrichment in the plasmid libraries to obtain MFEs. To avoid zeros in the numerator, a coverage-scaled pseudocount of 1 was added to the count of each mutation. Additionally, all mutations that were not observed at least 5 times in the mutagenized libraries were omitted from analysis.

To normalize the MFE scores across regions and between the two different viruses, we normalized each dataset to the MFE score of synonymous mutations, assuming these would have a neutral fitness score [Log2(syn MFE) = 0]. For the CVB3 libraries, since synonymous mutations were present throughout the proteome, the MFEs of each replicate were standardized to the average of all synonymous MFEs observed. These normalized MFEs were averaged across replicates to calculate the average MFE for each mutation, as long as at least two MFE values were observed among the independent replicates. Site MFEs were calculated as the average mutation MFE per site for each replicate, and averaged across replicates to calculate the average MFE for each site, as long as at least two site MFE values were observed among the independent replicates. As synonymous mutations were not introduced throughout the original EVA71 libraries, a sublibrary containing a synonymous mutation (642 GAA→GAG) and a representative mutagenized region from each of the two original libraries (sites 577:610 and 863:910 of capsid and replication libraries, respectively) was used for normalization. MFEs for this sublibrary were calculated and normalized to synonymous mutations as performed for the CVB3 libraries. A linear model was obtained between the synonymous-normalized MFEs of the sublibrary and the non-normalized MFEs of the corresponding subset of capsid and replication mutations. This model was then applied to the full dataset, enabling normalization across regions and standardization to synonymous mutation MFE in both viruses.

### Protein structure analyses

The EVA71 capsid structure was obtained from the PDB: 8E2X. The CVB3 capsid structure was based on the PDB: 4GB3, but modified to match the Nancy sequence as previously reported^49^. The CVB3 3D structure used was PDB: 3CDW. Structure prediction for all remaining CVB3 and EVA71 proteins was performed using the AlphaFold (version 3) server (https://alphafoldserver.com/)^51^. The structures of 2C and 3D from PV1 and EVD68 were predicted using AlphaFold3, based on the individual protein sequences extracted from the full polyproteins in UniProt entries P03300 and Q68T42, respectively. We used AlphaFold3 to model the interactions between the EVA71 and CVB3 3A proteins and human GBF1 (UniProt entry: Q92538). Secondary structure predictions were obtained from STRIDE^52^. Surface residues of the capsid and 2A were identified using the findSurfaceRes script in PyMOL with a cutoff of 2.5 Å^2^. For the capsid, outer surface residues were defined as those located more than 130 Å from the center of the capsid. Receptor footprints were obtained using the interfaceResidues script in PyMOL, with a cutoff of 3 Å to detect capsid residues in contact with the receptor in the PDB structures 6I2K (EVA71-SCARB2), 7VYK (CVB3-CAR), and 7VY5 (CVB3-DAF). The locations of the Walker motifs were extracted using a publication that describes the structure of the CVB3 2C protein^53^. Structures were colored by the site MFE difference attribute using the Render by Attribute tool in UCSF ChimeraX^54,55^. Capsid assembly interfaces between adjacent protomers and pentamers were identified using the select contact function in UCSF ChimeraX. Interacting surfaces were defined by having a buried solvent accessible surface area of 15 Å^2^ or greater. To determine the transmembrane topology of the 3A viral proteins from CVB3 and EVA71, their AA sequences were uploaded to the DeepTMHMM^38^ (version 1.0.44) web server (https://dtu.biolib.com/DeepTMHMM/). Domains of 3A (N-terminal, membrane-binding, and C-terminal domains) were obtained from UniProt annotations and published work (EVA71^44^ and UniProt entry P03313 for CVB3).

### Structural alignments and per-protein comparison

EVA71 and CVB3 proteins were structurally aligned in PyMOL using the align command to perform pairwise alignments based on sequence and structural similarities. Gaps in the alignments were excluded from subsequent analyses. To compare the two datasets, a normalization per protein was performed to account for differences in the two datasets, such as differences in sequencing coverage. For this, site MFEs were standardized using the formula below so that the average corrected MFE of each protein would be 0, and positive and negative scores would indicate sites with MFE above or below the average MFE of that particular protein, respectively.

### Formula 1.

For each site *i* of a protein *p*:

$$\text{corrected}\;\text{MFEi}=\frac{\text{MFEi}-min(\text{MFEp})}{\text{mean}(\text{MFEp})-\text{min}(\text{MFEp})}$$

### Natural diversity analyses

For examination of variability in natural sequence alignments, all available sequences matching the criteria “Enterovirus A”, “Enterovirus B”, “Enterovirus C”, or “Enterovirus D” were downloaded from NCBI virus into four separate FASTA files on September 26. 2024. Sequences marked as being lab-passaged or vaccine strains were excluded. Full-length polyprotein open reading frames were then identified and extracted using EMBOSS (Version 6.6.0), before being clustered at 98% similarity with cd-hit (Version 4.8.1)^56^. The Enterovirus A and B sequence sets were then combined so they could be aligned against one another. Sequences were first codon aligned using RevTrans 2.0^57^, then translated to AA sequences using the transeq function from EMBOSS^58^ and re-aligned using MAFFT^59^ (Version 7.505). The AA alignment was indexed to the EVA71 and CVB3 DMS WT strains using custom R code. For Shannon entropy and similarity calculations, Enterovirus A, Enterovirus B, EVA71, and CVB3 subalignments were extracted.

To calculate Shannon entropy, AA counts at each position were summed using the FreqMat function from the QSutils^60^ package (Version 1.20.0), then the formula below was used, where p is the frequency of a given AA at a given site.


ShannonEntropy=−∑p(x)log(p(x))

To assess how similar EVA71 and CVB3 are to one another and other Enterovirus A and B sequences, the DistanceMatrix function from the DECIPHER package^61^ (Version 2.30.0) was used. Pairwise percent dissimilarities were calculated between all sequences in the combined Enterovirus A and B alignment, then the results were filtered to the desired comparison (i.e. EVA71 vs. CVB3 sequences). Percent similarity is simply one minus percent dissimilarity. Percent similarities at the protein, capsid, and replication protein levels were determined using the same procedure on the appropriate alignment fragment.

Phylogenetic trees were constructed with RAxML^62^ (Version 8.2.12) and visualized in TreeViewer^63^ (Version 2.2.0). The ICTV representative AA sequence for each species was used for all species except Enterovirus A and B, for which the EVA71 and CVB3 DMS WT sequences were used instead. Trees were constructed using the PROTGAMMAWAG model and bootstrapped 1000 times.

### Phydms analysis

Phydms^39^ was used to evaluate if the incorporation of MFE into phylogenetic models could improve model fit compared to standard models and to calculate differential AA preferences between the DMS datasets and natural variation. For this, EVA71 and CVB3 sequences, or EVA and EVB sequences, were aligned and processed as indicated above for entropy measurements and split into the capsid (P1) and replication (P2, P3) regions. MFE values were transformed into site preferences by dividing each MFE value by the sum of all MFE values at each site. Phydms was then run using the default setting following preparation of the sequence alignment with the phydms_prepalignment function.

### Drug pocket prediction

Identification of druggable pockets was performed using the Schrödinger software (Version 13.8). Proteins were first subjected to the protein preparation workflow in Maestro using the default settings, and then SiteMap was used to define druggable pockets using the default settings, with sites having a SiteMap score >0.8 considered as druggable according to previously suggested criteria^64^. Homologous pockets were defined as those sharing more than 50% of their residues, relative to the size of the smaller pocket.

## Supplementary Material

1
