# Supplementary material for: Comparative Analysis of Deep Mutational Scanning Datasets in Enteroviruses A and B Identifies Functional Divergence and Therapeutic Targets
Source: Res Sq. 2025 Sep 8:rs.3.rs-7483105. Preprint. [Version 1] doi: 10.21203/rs.3.rs-7483105/v1 (PMC12440074; doi:10.21203/rs.3.rs-7483105/v1)
Supplement: 1 [file NIHPPRS7483105V1-supplement-1.pdf]

Supplementary material

A

Characteristics of DMS libraries

	Region	Mutagenesis method	del	syn
**CVB3 DMS plasmid libraries**	P1 region<br>P2 region<br>P3 region	PCR-based<br>synthetic oligos<br>synthetic oligos	-<br>+<br>+	+<br>+<br>+
**EVA71 DMS plasmid libaries**	P1 region<br>P2-P3 region	synthetic oligos<br>synthetic oligos	+<br>+	-<br>-
**EVA71 DMS plasmid sub-libary**	P1 (577:610)<br>P2 (863:910)	synthetic oligos	+	+

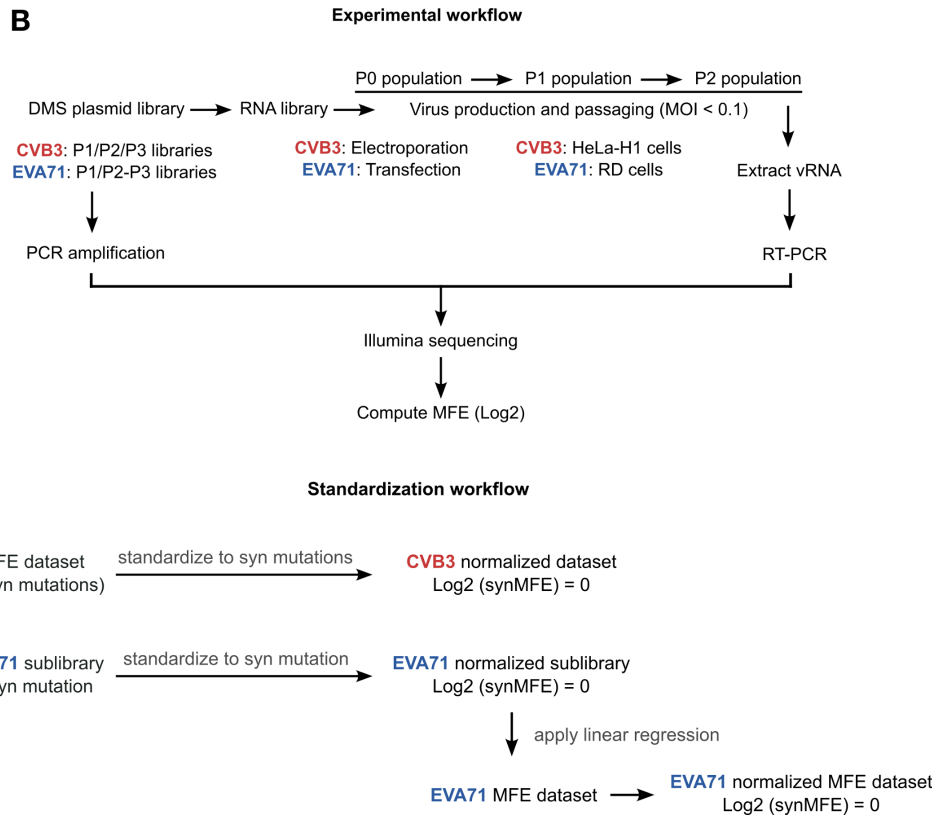

Supplementary figure 1. Overview of DMS library characteristics, experimental setup, and data processing. A) *Overview of DMS library characteristics, including regions mutagenized per library, mutagenesis method, and incorporation of deletions (del) and/or synonymous mutations (syn).* ***B)*** *Experimental workflow to produce mutagenized virus populations from DMS plasmid libraries and measure changes in mutation frequency between the initial plasmid populations and passage 2 viral populations.* ***C)*** *Summary of analysis steps to standardize MFEs to Log2(synMFE) = 0. For CVB3, genome-wide synonymous mutations enabled direct normalization. For EVA71, where synonymous mutations were not introduced, we used a representative sub-library containing a synonymous mutation and overlapping mutagenized regions. A linear model was fit between the normalized MFEs of this sub-library and the corresponding unnormalized regions, and applied to the full dataset.*

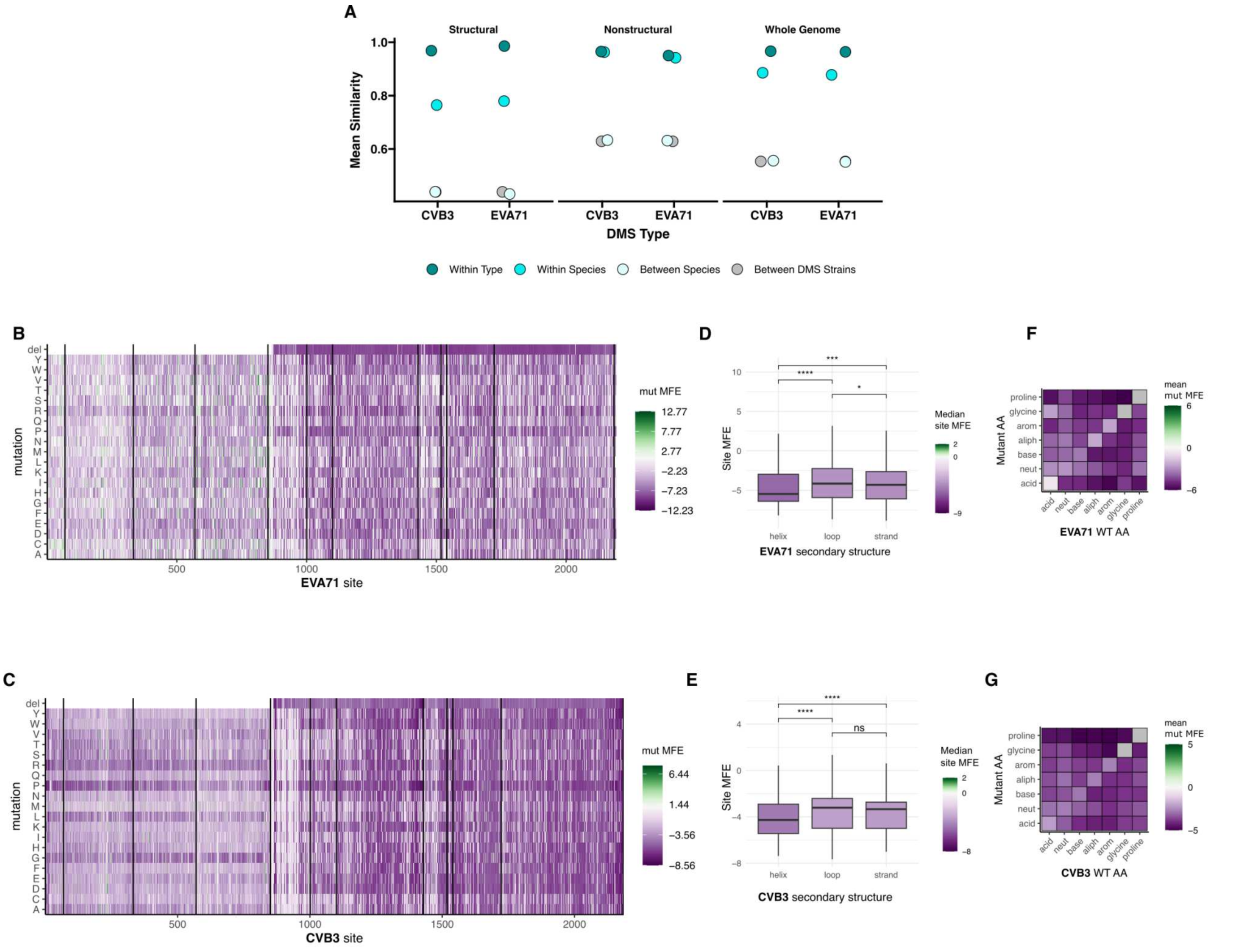

Supplementary figure 2. Evolutionary similarity and distribution of MFEs in EVA71 and CVB3. A) *Similarity per proteome region at different evolutionary levels.* ***B)*** *Heatmap of MFEs across EVA71 proteins.* ***C)*** *Heatmap of MFEs across CVB3 proteins.* ***D)*** *Boxplots of mean site MFE by predicted secondary structure elements in CVB3.* ***E)*** *Boxplots of mean site MFE by predicted secondary structure elements in EVA71. Statistical significance in (D) and (E) was calculated using a Wilcoxon test.* ***F-G)*** *Mean MFE for each type of AA substitution, categorized by WT and mutant residue class in EVA71 (F) and CVB3 (G).*

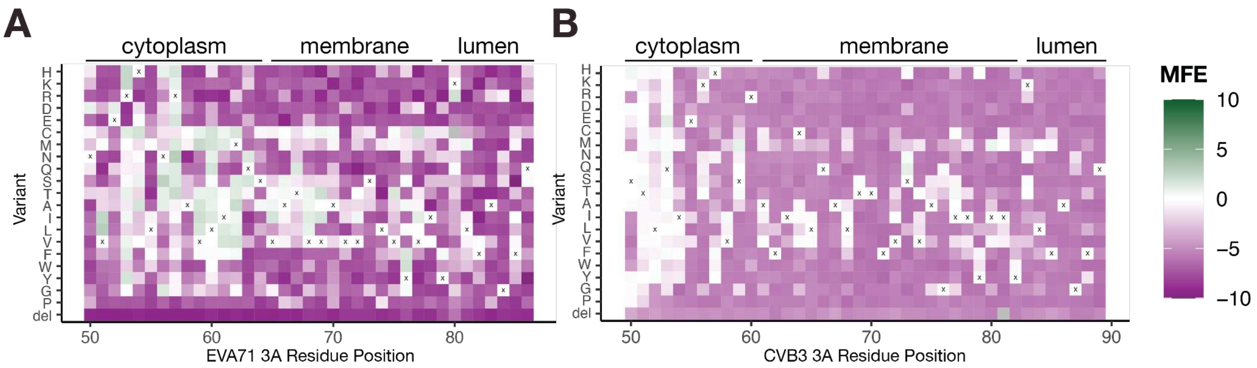

Supplementary figure 3. Mutational tolerance at the 3A membrane anchor. A, B) *Heatmaps displaying the MFE scores of mutations within the C-terminal membrane-binding domain of EVA71 (A) and CVB3 (B) 3A. Predicted protein topology (cytoplasm, membrane, or lumen) was determined by DeepTMHMM. The "X" symbol indicates WT AA position.*

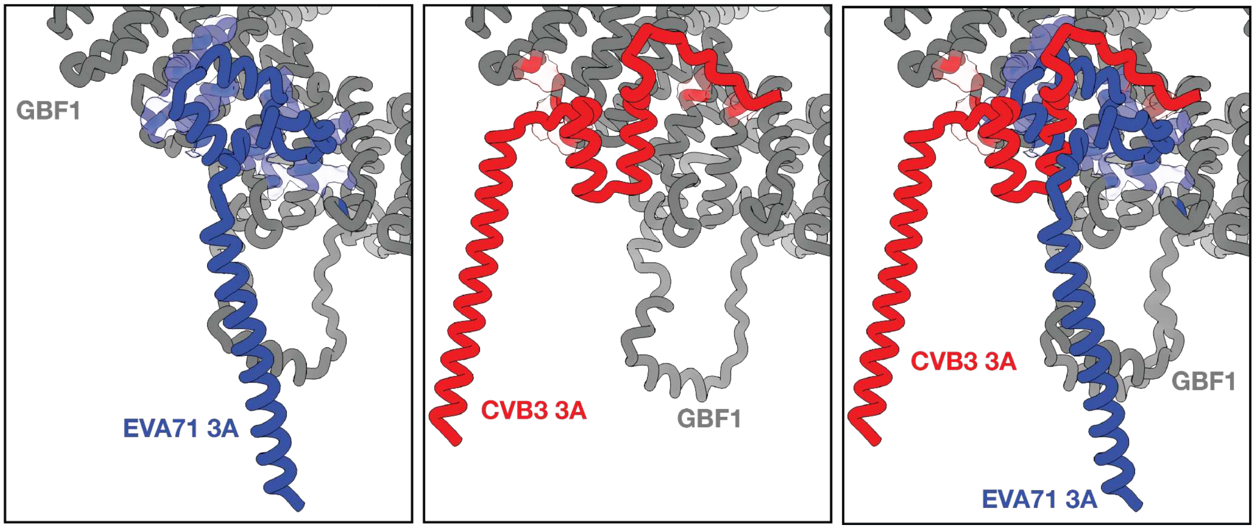

Supplementary Figure 4. EVA71 and CVB3 3A-GBF1 Interaction Modes *Structural model of the interaction interface between the viral 3A protein (from EVA71 and CVB3) and the host factor GBF1. A semi-transparent surface representation of GBF1 highlights the residues that are in contact with the viral 3A protein.*

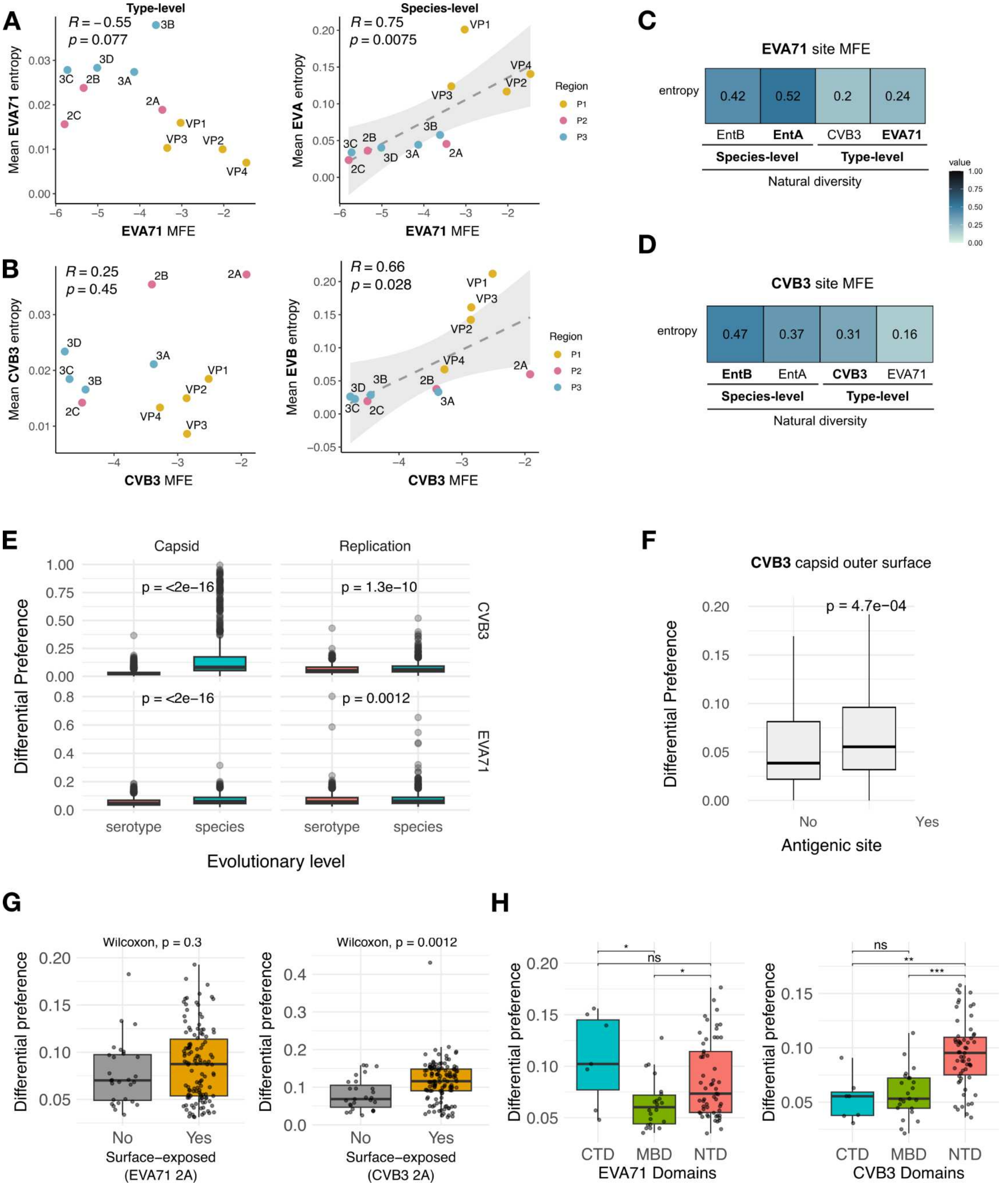

Supplementary figure 5. Comparison of experimental MFEs and natural variation. A-B) *Correlation of average site MFE per protin and natural variation (average Shannon entropy) at different evolutionary levels for EVA71 (A) and CVB3 (B).* ***C-D)*** *Correlation of site MFE and natural variation (Shannon entropy) at different evolutionary levels for EVA71 (C) and CVB3 (D).* ***E)*** *Distribution of differential preference by functional region at different evolutionary levels for EVA71 and CVB3 .* ***F)*** *Differential preferences between DMS datasets and natural sequences at the type level in known antigenic sites versus non-antigenic surface sites in the CVB3*

capsid. ***G)*** *Differential preferences between DMS datasets and natural sequences at the type level in surface-exposed versus burried sites for EVA71 2A and CVB3 2A.* ***H)*** *Differential preferences between DMS datasets and natural sequences at the type level in the different structural domains of EVA71 3A and CVB3 3A. Statistical significance was calculated using a Wilcoxon test. For 3A, adjusted p-values are calculated using the Benjamini-Hochberg (BH) method.*

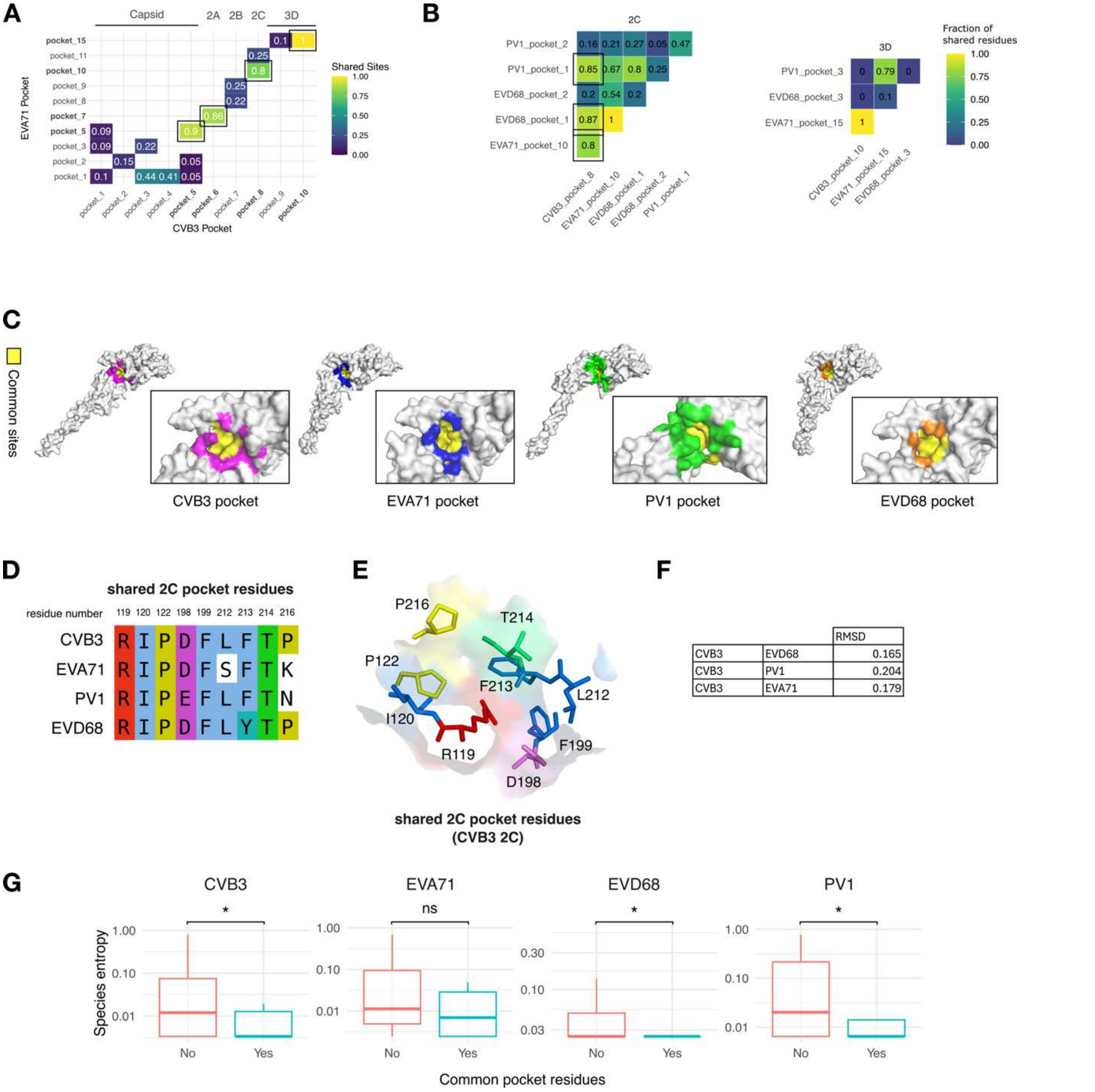

Supplementary Figure 6. Conservation of drug pockets across human enteroviruses. A) *Fraction of shared residues per pocket between EVA71 and CVB3.* ***B)*** *Fraction of shared residues per pocket across EVA71, CVB3, PV1, and EVD68, limited to pockets found in proteins 2C and 3D. In panels A and B, fractions are calculated relative to the smallest pocket being compared.* ***C)*** *Mapping of residues forming the 2C pocket in each virus. The 9 residues conserved across all viruses based on sequence alignments are highlighted in yellow.* ***D)*** *Shared residues in the pan-enterovirus 2C pocket based on sequence alignments, colored by physicochemical AA properties.* ***E)*** *Residues in the CVB3 2A pocket that are shared with 2C pockets in the other viruses, colored by physicochemical AA properties.* ***F)*** *Root-mean-square deviation (RMSD) of the 9 conserved 2C pocket residues in EVD68, PV1, and EVA71, compared to those in the CVB3 2C pocket.* ***G)*** *Entropy from natural sequence alignments for the 9 conserved 2C pocket residues compared to residues in the rest of the proteome.*

Virus	Evolutionary level	Proteome region	Model	deltaAIC	LogLikelihood	nParams	ParamValues
EVA71	Type	Capsid	ExpCM_capsid_prefs	0.00	-15473.31	6	beta=1.00, kappa=13.87, omega=0.03
EVA71	Type	Capsid	averaged_ExpCM_capsid_prefs	1985.80	-16466.21	6	beta=0.00, kappa=13.80, omega=0.01
EVA71	Type	Capsid	YNGKP_M5	1994.14	-16464.38	12	alpha_omega=0.30, beta_omega=10.00, kappa=13.71
EVA71	Type	Capsid	YNGKP_M0	2076.84	-16506.73	11	kappa=13.57, omega=0.02
EVA71	Type	Replication	ExpCM_replication_prefs	0.00	-36868.57	6	beta=0.86, kappa=6.90, omega=0.03
EVA71	Type	Replication	YNGKP_M5	1598.70	-37661.92	12	alpha_omega=0.30, beta_omega=9.28, kappa=6.03
EVA71	Type	Replication	averaged_ExpCM_replication_prefs	2397.32	-38067.23	6	beta=0.24, kappa=7.08, omega=0.02
EVA71	Type	Replication	YNGKP_M0	2448.50	-38087.82	11	kappa=5.95, omega=0.02
CVB3	Type	Capsid	ExpCM_capsid_prefs	0.00	-19761.40	6	beta=2.24, kappa=8.78, omega=0.09
CVB3	Type	Capsid	YNGKP_M5	4115.40	-21813.10	12	alpha_omega=0.30, beta_omega=10.00, kappa=8.43
CVB3	Type	Capsid	averaged_ExpCM_capsid_prefs	4185.10	-21853.95	6	beta=0.66, kappa=8.84, omega=0.01
CVB3	Type	Capsid	YNGKP_M0	4276.52	-21894.66	11	kappa=8.40, omega=0.01
CVB3	Type	Replication	ExpCM_replication_prefs	0.00	-43867.01	6	beta=1.30, kappa=5.18, omega=0.03
CVB3	Type	Replication	YNGKP_M5	3249.84	-45485.93	12	alpha_omega=0.30, beta_omega=10.00, kappa=4.46
CVB3	Type	Replication	averaged_ExpCM_replication_prefs	3653.44	-45693.73	6	beta=0.00, kappa=5.04, omega=0.02
CVB3	Type	Replication	YNGKP_M0	3986.78	-45855.40	11	kappa=4.40, omega=0.02
EVA71	Species	Capsid	ExpCM_capsid_prefs	0.00	-18020.80	6	beta=0.87, kappa=2.29, omega=0.05
EVA71	Species	Capsid	YNGKP_M5	1553.42	-18791.51	12	alpha_omega=0.32, beta_omega=10.00, kappa=2.41
EVA71	Species	Capsid	YNGKP_M0	2579.32	-19305.46	11	kappa=2.34, omega=0.03
EVA71	Species	Capsid	averaged_ExpCM_capsid_prefs	2586.04	-19313.82	6	beta=0.49, kappa=2.35, omega=0.03
EVA71	Species	Replication	ExpCM_replication_prefs	0.00	-52884.08	6	beta=0.95, kappa=4.39, omega=0.04

EVA71	*Species*	*Replication*	*YNGKP_M5*	*1908.50*	*-53832.33*	*12*	*alpha_omega=0.30, beta_omega=10.00, kappa=3.90*
EVA71	*Species*	*Replication*	*averaged_ExpCM_replication_prefs*	*3672.92*	*-54720.54*	*6*	*beta=0.03, kappa=4.34, omega=0.03*
EVA71	*Species*	*Replication*	*YNGKP_M0*	*3794.34*	*-54776.25*	*11*	*kappa=3.85, omega=0.02*
CVB3	*Species*	*Capsid*	*ExpCM_capsid_prefs*	*0.00*	*-87834.73*	*6*	*beta=1.70, kappa=1.89, omega=0.08*
CVB3	*Species*	*Capsid*	*YNGKP_M5*	*2815.04*	*-89236.25*	*12*	*alpha_omega=0.39, beta_omega=10.00, kappa=2.11*
CVB3	*Species*	*Capsid*	*YNGKP_M0*	*14634.84*	*-95147.15*	*11*	*kappa=1.96, omega=0.02*
CVB3	*Species*	*Capsid*	*averaged_ExpCM_capsid_prefs*	*15009.40*	*-95339.43*	*6*	*beta=1.13, kappa=2.07, omega=0.03*
CVB3	*Species*	*Replication*	*ExpCM_replication_prefs*	*0.00*	*-99797.71*	*6*	*beta=1.33, kappa=4.73, omega=0.03*
CVB3	*Species*	*Replication*	*YNGKP_M5*	*3485.60*	*-101534.51*	*12*	*alpha_omega=0.30, beta_omega=6.96, kappa=4.07*
CVB3	*Species*	*Replication*	*averaged_ExpCM_replication_prefs*	*5684.18*	*-102639.80*	*6*	*beta=0.00, kappa=4.56, omega=0.02*
CVB3	*Species*	*Replication*	*YNGKP_M0*	*6715.74*	*-103150.58*	*11*	*kappa=4.03, omega=0.02*

Supplementary Table 1. Results of the phydms analysis for each region of the EVA71 and CVB3 proteomes using phylogenetic models at the type or species levels*. Models shown include: YNGKP M0 and M5, variants of the Yang–Nielsen–Goldman–Kumar–Pamilo (YNGKP) codon-substitution models; ExpCM, an experimentally informed codon model that integrates DMS AA preferences to model selection at each site; Control ExpCM, a version of the ExpCM in which experimental preferences are averaged across all sites, rendering the model non-site-specific, thus serving as a control to assess the significance of incorporating site-specific DMS data. Beta reflects how strongly natural evolution adheres to the site-specific amino-acid preferences inferred from DMS experiments. A $\beta > 1$ implies that selection in nature prefers the same AA but with a greater stringency, while $\beta < 1$ suggests lower stringency in nature. Omega represents the relative rate of nonsynonymous versus synonymous substitutions after accounting for site-specific AA preferences. Kappa indicates the transition/transversion rate ratio governing nucleotide substitutions in the codon models. Model performance is evaluated using ΔAIC, with lower values indicating improved model fit.*
